# Therapeutic and Formulation Advances of Ivermectin in Veterinary and Human Medicine

**DOI:** 10.3390/pharmaceutics17111384

**Published:** 2025-10-25

**Authors:** Nicezelle Gernandt, Chanri Wentzel, Daniélle van Staden, Wilna Liebenberg, Hendrik J. R. Lemmer, Minja Gerber

**Affiliations:** Centre of Excellence for Pharmaceutical Sciences (Pharmacen™), North-West University, Potchefstroom 2531, South Africa; 25834126@mynwu.ac.za (N.G.); chanriwentzel@gmail.com (C.W.); dvanstaden711@gmail.com (D.v.S.); wilna.liebenberg@nwu.ac.za (W.L.); righard.lemmer@nwu.ac.za (H.J.R.L.)

**Keywords:** antibacterial, antiparasitic, antiviral, dosage form, drug repurposing, human, infectious disease, ivermectin, veterinary, targeted drug delivery

## Abstract

The treatment of parasitic infections has evolved in terms of effectiveness and the prevention of drug resistance. This is highlighted by the discovery of ivermectin (IVM), a macrocyclic lactone and broad-spectrum antiparasitic agent. IVM garnered scientific attention by presenting a therapeutic alternative in the field of veterinary medicine due to its control of multiple parasite species, including nematodes and soil-transmitted helminths. Shortly after its discovery, IVM was approved for human use by the World Health Organization (WHO) and United States Food and Drug Administration (FDA) for combating head lice, onchocerciasis, rosacea, scabies, and worm infestations within the gastrointestinal tract (GIT). In veterinary medicine, IVM is available in a range of formulations and can be administered via different routes (i.e., oral, topical, and parenteral), whereas for humans, IVM is only approved as a single oral dose and dermal cream. Establishing a comprehensive overview of IVM’s applications in both human and veterinary medicine is necessary, particularly in light of its repurposing potential as a treatment for various conditions and emerging diseases. Given its primary application in veterinary medicine, there is a need to enhance the development of dosage forms suitable for human use. Therefore, this review details the discovery, mechanisms, and applications of IVM, while also examining the challenges of resistance, side-effects, and controversy surrounding its use, to ultimately emphasize the importance of targeted, optimized IVM delivery via tailored dosage form development in animals and humans as part of the One Health approach to interlink innovations across veterinary and human medicine fields.

## 1. Introduction

Few pharmaceutical compounds have had an impact as profound within their field as ivermectin (IVM). Its effectiveness in combating parasitic infestations has transformed global animal and human health, particularly in regions burdened by neglected tropical diseases [1]. In this regard, IVM’s role in the treatment of parasitic diseases can be compared to that of penicillin during World War II, when penicillin significantly advanced the treatment of bacterial infections, leading to reduced mortality [2].

**Figure 1:**
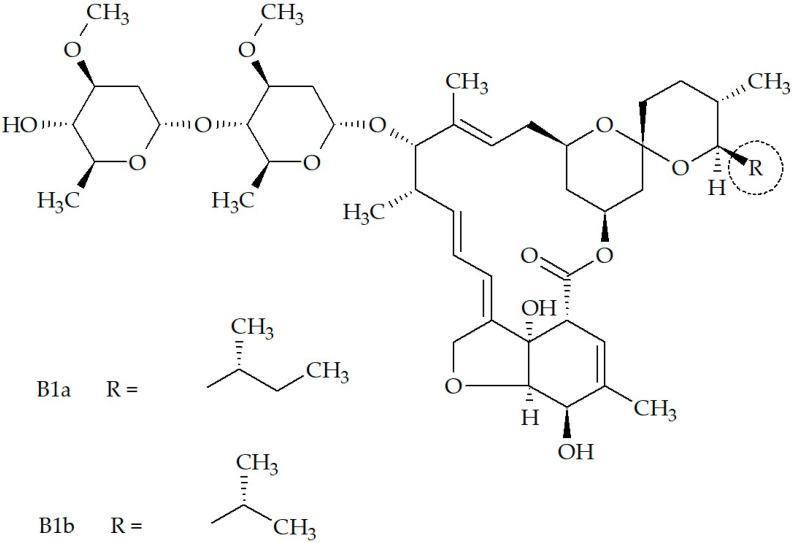
The chemical structure of IVM.

IVM, a semi-synthetic avermectin, was first discovered by Satoshi Õmura in the 1970s during the fermentation broth of the soil bacterium, *Streptomyces avermitilis* [3]. Thereafter, it underwent chemical modification leading to its synthesis (22,23-dihydroavermectin B1) [4] that produces a mixture containing two chemically altered avermectins, consequently containing ≥80% 22,23-dihydroavermectin B1a and ≤20% 22,23-dihydroavermectin B1b [5,6,7] (Figure 1).

Following its discovery, IVM was commercialized across various sectors, including the veterinary, agricultural and aquaculture divisions during 1981, thereby establishing the use of this highly hydrophobic molecule due to its superior efficacy and safety profile compared to other avermectins [7,8]. IVM was officially validated and registered for human use six years later for the treatment of onchocerciasis [7] with dosage forms intended for oral ingestion and/or topical skin administration [7,9].

Avermectins are described as macrocyclic lactones, generally exhibiting potent anthelmintic properties [8], which have evolved the treatment regimens of parasitic diseases in both animals and humans. These compounds act by binding to glutamate-gated chloride channels found in the nerve and muscle cells of invertebrates, causing paralysis and death of the parasite [10,11,12]. This mechanism of action exhibits high selectivity, as the targeted chloride channels are not present in vertebrates, enabling effective elimination of parasitic infestations while minimizing toxicity towards animal and human hosts [10,11,12]. The avermectin family includes several closely related compounds, including abamectin, doramectin, eprinomectin and IVM, each exhibiting slightly different pharmacological profiles and usage patterns [10,11,12]. However, IVM is the most widely recognized member, mainly due to its extensive use in veterinary and human medicine. IVM is also of particular importance for controlling neglected tropical diseases like onchocerciasis, strongyloidiasis and lymphatic filariasis in humans [10,11,12], thereby providing crucial aid in the roadmap established by the World Health Organization (WHO) to gain control of neglected tropical diseases by 2030 [13].

IVM possesses broad-spectrum microfilaricidal activity against filarial nematodes as well as endectocidal activity against *Trichuris trichiura* (whipworm), scabies and malaria. It is also the first line of treatment for *Strongyloides stercoralis* (roundworm) infections, onchocerciasis, and lymphatic filariasis, contributing to increased awareness and demand for its usage, especially since it aligns with ambitious public health goals for disease elimination [14]. In most cases, IVM does not possess the ability to kill adult parasites; however, it suppresses microfilarial reproduction, thereby impeding disease progression [15]. Furthermore, IVM exhibits antiparasitic activity in other parasite-induced diseases for which it is not part of the conventional treatment regimen, such as malaria, trypanosomiasis, schistosomiasis, trichinosis, and leishmaniasis [16]. Additional studies have concluded that not only does IVM disrupt the neurophysiology of parasites, but it also influences the immune response of the host; thereby signifying that IVM develops a memory response mechanism within the host’s immune system, enabling the immune response to eliminate pathogens [17,18,19,20,21].

Nevertheless, the widespread and prolonged use of IVM has led to growing concerns about the emergence of drug-resistant parasites, which poses a significant threat to its continued efficacy. Since its introduction, IVM resistance has developed relatively quickly in several veterinary parasite populations and is now widely documented [22,23,24,25,26,27,28,29,30,31,32,33]. A consequence of the mass drug administration approach using IVM to protect animals at risk of infection is the emergence of IVM-resistant parasite strains [25]. Parasite control strategies are also hindered by the lack of understanding regarding the underlying mechanisms of action of IVM, as well as a gap in knowledge regarding diagnostic resistance markers [25,32]. Therefore, optimized formulation strategies and targeted administration methods of antiparasitic agents like IVM play a critical role in influencing the development and spread of drug resistance [34]. For instance, dosage forms that ensure sustained therapeutic drug concentration levels are of particular importance in antiparasitic therapy, as subtherapeutic exposure can allow partially resistant parasites to survive and propagate within the host [35].

Long-acting injectable formulations or slow-release boluses, commonly used in veterinary practice, maintain plasma drug concentrations above the minimum effective level for extended periods, reducing the opportunity for resistant parasites to emerge [36]. Moreover, topical or locally acting formulations incorporating IVM, used in both human and veterinary medicine, enable high drug concentrations at the site of infection while limiting systemic exposure and avoiding the elimination of non-targeted organisms needed to maintain a healthy microbiome [37]. User-friendly dosage forms can aid in resistance prevention by improving therapeutic adherence. For instance, single-dose or long-acting formulations reduce the likelihood of missed treatments, which can otherwise promote resistance [36]. Additionally, targeted delivery systems that concentrate IVM in specific tissues or parasite niches may enhance efficacy and improve selectivity, restricting toxic effects on non-target organisms [37]. In some cases, combination therapy incorporating IVM with other antiparasitic agents may impede resistance by simultaneously targeting multiple pathways within the parasite, though this approach is more commonly explored in human medicine [38,39,40], whereas in livestock, controlled-release dosage forms may limit environmental shedding of subtherapeutic drug residues, which can contribute to resistance among free-living stages of parasites or environmental microbial communities [41,42].

Formulation strategies are increasingly recognized as valuable tools in managing and potentially impeding the development of IVM resistance, since novel formulations have the potential to modify the pharmacokinetic characteristics of IVM in a manner that increases its therapeutic efficacy while simultaneously lowering the risk of resistance development via targeted drug delivery [43,44,45,46,47]. Thus, this review will provide an updated overview of established and novel therapeutic indications for IVM, its toxicity, existing veterinary dosage forms comprising IVM, progress involving in vivo translation studies, existing human dosage forms incorporating IVM, and recent developments of novel IVM-containing dosage forms.

Ivermectin is regulated under separate, purpose-built frameworks for humans and animals. Human regulation (e.g., by FDA CDER) mandates extensive clinical trials for specific parasitic diseases and post-market surveillance. Veterinary regulation (e.g., by FDA CVM) prioritizes target animal safety, efficacy, and environmental impact, with additional requirements for food-producing animals, such as residue studies to set withdrawal periods and safe meat/milk levels [48]. These fundamental differences, centered on human patients versus animal welfare, environment, and consumer food safety, indicate that veterinary and human formulations are not interchangeable [49].

## 2. Veterinary Uses

### 2.1. Established

**Figure 2:**
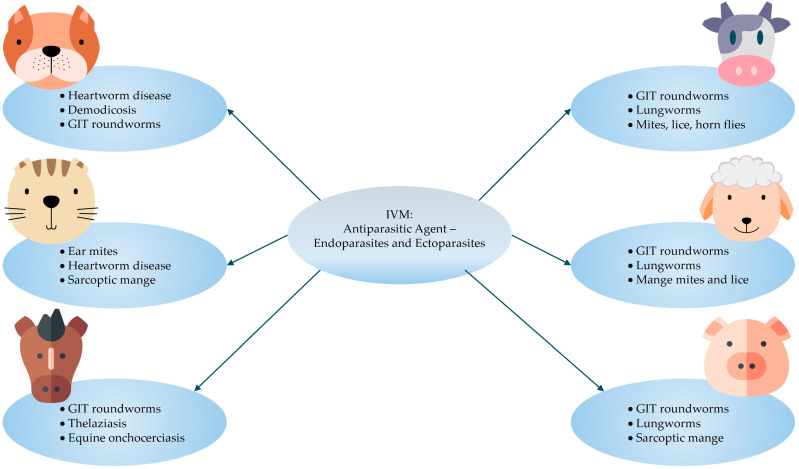
Therapeutic indications of IVM in veterinary medicine (icons designed by Freepik).

A summary of the indications for IVM therapy in animals is illustrated in Figure 2. According to Martin et al. [50], IVM shows potent anthelmintic and insecticide activity when it was administered via the oral route at a therapeutic dose of 150–200 µg/kg. The established therapeutic dosage ranges for IVM use in different animals [51] are listed below:

1. Small animals: 0.300–0.600 mg/kg (oral dose, once daily, until two respective negative skin scrapings are obtained one month apart).
2. Cattle and sheep: 0.200 mg/kg (single dose subcutaneous injection).
3. Horses: 0.200 mg/kg (oral dose, repeated as necessary for adequate parasite management).
4. Swine: 0.300 mg/kg (subcutaneous injection, repeated two-weekly) or 0.100–0.200 mg/kg (oral dose in feed for seven days).

Alternatively, it can be premixed with food at a dosage of 100 μg/kg/day for 7 days, followed by a second administration within 21 days of the initial treatment (for example, 7 days treatment, 7 days non-treatment, 7 days treatment). This treatment method has shown optimal results in eradicating herd infestations [52]. The recommended treatment regimen for heartworm disease in dogs and cats is 200 µg/kg of IVM, administered as an oral dose [53]. Similarly, a 200 µg/kg dose (in a range of different types of formulations) was successful in the prevention and treatment of intestinal roundworm infestations in livestock (cattle, goats, sheep, pigs, and horses) [1,54]. IVM can also be used as a dewormer for domestic animals, which is purchasable as a tablet, chewable tablet, topical liquid, and subcutaneous injection [55].

### 2.2. Investigational

Experimental data exhibited controlled-release capsules containing IVM successfully eradicated nematode infestations when administered to sheep [1,56]. The results published by Forbes et al. [57] showed total parasitic elimination and prevention of infestation with the intraruminal injection of a controlled-release (long-acting) formulation at a dose of 20–40 µg/kg per day for a period of 100 days in sheep suffering from mange disease. IVM also demonstrated efficiency against mange infection in cattle and pigs at varying doses and administration routes [58,59,60,61].

Particularly intriguing is the comparison of disposition kinetics of IVM in cattle and pigs, where pigs exhibited lower systemic availability of IVM compared to cattle following subcutaneous injections. This interspecies variability in pharmacokinetic behavior may be attributed, in part, to differences in anatomical composition and tissue distribution patterns. For example, pigs tend to have a larger proportion of adipose tissue, which can function as a reservoir for lipophilic compounds such as IVM, thereby reducing its immediate plasma availability [52]. This depot effect arises due to IVM’s high lipophilicity, which favors partitioning into lipid-rich tissues, resulting in prolonged retention and slower systemic release [62]. Consequently, in certain therapeutic contexts, such as the treatment of sarcoptic mange in pigs, repeated administration on day seven post-initial dosing may be necessary to achieve and maintain therapeutic drug levels [52]. On the other hand, the accumulation of IVM in adipose tissue raises concerns regarding potential toxicity, particularly with frequent or high-dose regimens, as sustained tissue retention may lead to delayed systemic clearance and prolonged exposure. Addressing this challenge through the development of optimized dosage forms, such as sustained-release formulations (e.g., solid lipid dispersions) [63], targeted delivery systems (e.g., solid lipid nanoparticles (SLNs) for transdermal drug delivery) [64,65,66] or parenteral lipid-based depot formulations [67], may enhance therapeutic efficacy while minimizing the risk of adverse effects associated with drug accumulation.

For cattle, IVM is regarded as one of the most efficient therapies for psoroptic mange, which is a severe dermatological condition caused by infestations of parasitic mites. Even though it does not affect mite eggs, a single 0.2 mg/kg injection can eliminate *Psoroptes ovis* infestations and the continued presence of therapeutic IVM concentrations within the treated animal ensures the death of newly hatched larvae [52].

Cattle and horses suffering from thelaziasis (eye worm infestation induced by nematodes) may display different degrees of ocular inflammation along with a range of symptoms, which can lead to blindness [68,69,70]. A topical pour-on formulation with a dose of 1.0 mL/kg proved to be effective in eliminating such eye infections. Additionally, a 200 µg/kg subcutaneous injection of IVM entirely purges cattle of nematode infections [68].

Other studies reported that oral IVM administration in the form of a paste (200 µg/kg) was used to cure ponies with lungworm infections [1,71]. Both an injection and an orally injectable paste formulation (each containing 200 µg/kg of IVM) also showed effective antiparasitic action against microfilariae in equine onchocerciasis [72,73]. Microfilariae of adult worms from the *Onchocerca* species cause infection in equine onchocerciasis, transmitted via various fly species’ bites [70]. Clinical presentation of equine onchocerciasis includes pruritus, alopecia, and dermatitis [72].

Both oral and subcutaneous administration of IVM (at a dose of 200 µg/kg) were successful as therapeutic modes of treatment and prevention of lice infestation in livestock [1,74]. Compared to oral and topical treatments, the subcutaneous route demonstrated superior efficacy against internal and external parasites in terms of drug bioavailability in sheep, cattle, and goats [75]. However, when considering lice infestations, the topical treatment (pour-on formulations) of IVM has proven more effective than subcutaneous treatments against biting lice, since higher IVM concentrations are achieved within the skin after topical administration, and the lice then come in direct contact with IVM and ingest it orally, resulting in their death. Moreover, during subcutaneous treatment, the IVM dose is divided between the skin and bloodstream (plasma availability) rendering lower doses available at the site of infection (skin) [52].

### 2.3. Animal Toxicity Investigations and Applications

In vivo studies play a critical role during the evaluation of pharmacological agents, offering essential insights into their efficacy, safety, pharmacokinetics, and mechanisms of action under physiologically relevant conditions [76,77]. Unlike in vitro experiments, which are limited to controlled laboratory environments, in vivo models allow for the observation of complex biological interactions within whole organisms [76,77]. These studies are indispensable for bridging the gap between preclinical research and clinical application, particularly in assessing therapeutic potential and predicting human responses [76,77]. The collection of evidence from in vivo investigations has been instrumental in supporting the repurposing, dosage form optimization and expanded use of IVM across a variety of species and disease contexts.

A study conducted by Madrid et al. [78] investigated the repurposing of IVM by formulating it into an oral powder dosage form by means of freeze-drying. The study also included an in vivo evaluation of the safety and tolerability of high dosage oral IVM administration in Corydoras fish models. The key findings drawn from this study were that high doses of 0.22 and 0.86 mg/kg did not cause harm to intestinal tissues or affect blood cell counts, whereas an overdose of 170 mg/kg affected Myosin-Vb, which is one of the proteins involved in the movement of cells and intercellular transport of materials. The disruption in this protein thus potentially harms the intestinal epidermal integrity of the fish [78]. These findings provided valuable information on the safety of increased IVM doses, which is crucial for preventing drug resistance and exploring new therapeutic applications/dosage forms through drug repurposing.

An investigative study performed by Archana et al. [79] evaluated the degree of IVM toxicity in five healthy female goats following repeated subcutaneous administration for 14 days. The adverse effects caused by IVM during this treatment period were measured by monitoring various clinical parameters (respiratory rate, pulse rate and body temperature); hematological parameters (hemoglobin (Hb%), total leucocyte count (TLC) and differential leucocyte count (DLC)) and biochemical parameters (blood glucose, serum cholesterol, blood urea nitrogen (BUN), total plasma protein, alanine transaminase (ALT) and aspartate transferase (AST)). The study concluded that IVM induced no toxicities on clinical-, hematological- and biochemical parameters; however, slight increases in AST within acceptable physiological ranges were displayed. As such, careful consideration must be implemented when administering IVM in animals that are predisposed to neurological- and cardiac disorders. Finally, the normal physiology of the goat hosts was unaffected by IVM administration [79].

Al-Azzam et al. [80] assessed beagle dogs that were experimentally infected with *Brugia pahangi* and compared the plasma disposition kinetics of IVM and moxidectin after oral administration at a dose of 250 µg/kg. The results indicated that IVM achieved a peak plasma concentration (C_max_) of 132.6 ± 43.0 ng/mL, with a terminal elimination half-life (t_½_) of 80.3 ± 29.8 h. In contrast, moxidectin exhibited a higher C_max_ of 234.0 ± 64.3 ng/mL and a significantly longer t_½_ of 621.3 ± 149.3 h, highlighting differences in the pharmacokinetic profiles of these two drugs. Oral IVM in dogs resulted in moderate systemic exposure and a relatively short half-life compared to moxidectin. The oral route allowed for therapeutic plasma concentrations, but the differences in elimination half-lives suggest that drug formulation and lipophilicity significantly impact drug duration of action.

In a study involving Biłgorajska geese [81], IVM was administered at a dose of 0.2 mg/kg via intravenous and oral routes, respectively. The pharmacokinetic analysis revealed that after intravenous administration, IVM remained quantifiable in plasma up to 240 h, whereas after oral administration, it was detectable up to 144 h. The bioavailability following oral administration was approximately 20.38%, indicating limited absorption from the gastrointestinal tract. Intravenous injection led to sustained plasma levels over a longer duration compared to oral dosing, which showed lower bioavailability of around 20.4%. This indicates poor gastrointestinal absorption of IVM in geese, rendering the oral route of administration a less suited option when sustained systemic exposure is desired [81].

Shu and Okonkwo [82] conducted a study using rabbits, where IVM was administered subcutaneously at a dose of 150 µg/kg. The pharmacokinetic results encompassed a C_max_ of 34.0 ± 1.6 ng/mL, a duration to reach C_max_ (T_max_) of 1.4 ± 0.4 h, and a t_½_ of 10.4 ± 2.3 h. The study also noted a secondary peak in plasma concentration, suggesting the occurrence of enterohepatic recirculation. Subcutaneous injection led to rapid absorption and moderate systemic exposure, with evidence of enterohepatic recirculation extending IVM’s presence. This route allowed for efficient delivery and a pharmacokinetic profile suitable for parasitic control in lagomorphs.

Another study in rhesus macaques [83] administered high doses of oral IVM (30 or 60 mg) every third day to assess its pharmacokinetics in a primate model. The results indicated that repeated dosing did not lead to significant changes in the clearance or t_½_ of the drug, suggesting a lack of autoinhibition. However, the small sample size (three macaques per treatment group) warrants caution in interpreting these findings. Oral high-dose IVM was well tolerated and maintained consistent pharmacokinetic parameters with repeated administration. The oral route proved effective in achieving therapeutic concentrations without evidence of drug accumulation or metabolic saturation, supporting its feasibility for controlled dosing in primates.

These interpretations emphasize that the route of administration and dosage form significantly impact IVM’s pharmacokinetics, affecting absorption, bioavailability, and duration of action. This pharmacological variability provides important context when transitioning to IVM use in humans, where therapeutic efficacy, safety profiles and optimizing dosing regimens depend heavily on these factors. Given this foundation, the subsequent section will focus on the use of IVM in human medicine, examining its therapeutic roles, dosage forms, and clinical outcomes.

## 3. Human Uses

### 3.1. Established

**Figure 3:**
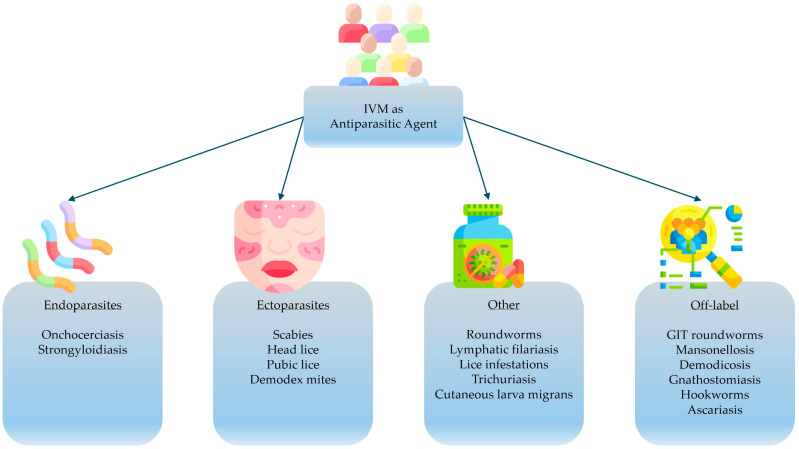
Therapeutic indications of IVM as antiparasitic agent in human medicine (Icons designed by Freepik).

**Figure 4:**
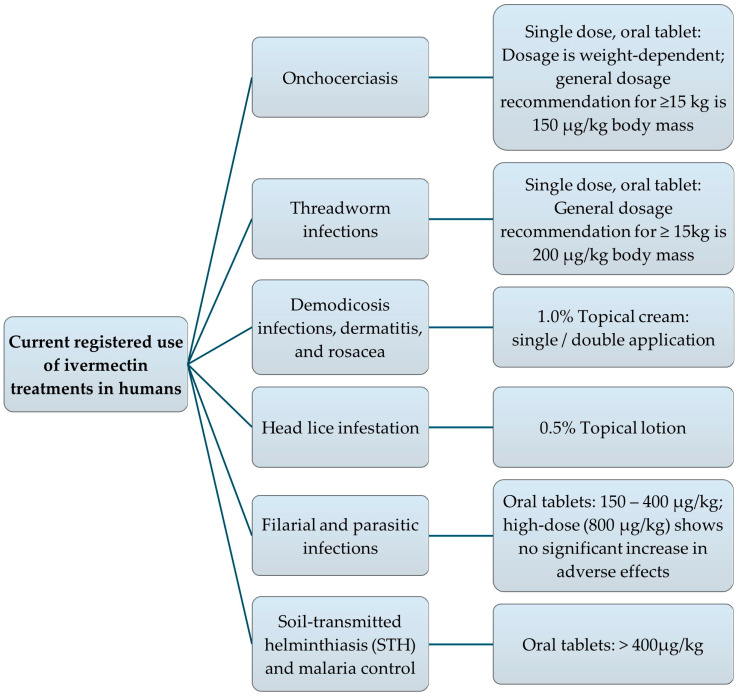
Schematic summary presenting the current approved (registered) therapeutic indications and dosage forms of ivermectin in humans.

Figure 3 and Figure 4, respectively, visualize a summary of the potential indications for administering IVM in humans, as well as the approved therapeutic indications and dosage forms for IVM consumption in humans. Among these IVM-based therapies, oral IVM (the most popular route of administration permitted for human use) is available in various dosage forms, including solutions, tablets, and capsules. However, the liquid solution dosage form provides double the systemic bioavailability of the solid dosage forms [17]. This increased oral bioavailability is largely attributed to the fact that IVM is already in a dissolved state when presented in the liquid dosage form, enabling enhanced drug permeability and thus absorption [84,85,86]. Consequently, formulating dosage forms that maximize drug solubility remains a key strategy for improving the bioavailability and consequent therapeutic efficacy of IVM [86,87,88].

According to Sharun et al. [64], numerous IVM formulations have been developed over time, including stable aqueous formulations, controlled release capsules, osmotic pumps, zein microspheres, silicone carriers, biodegradable microparticulate drug delivery systems, SLNs, lipid nanocapsules, solid dispersion suspension with sustained release of IVM, biodegradable subcutaneous implants and sustained-release varnish containing IVM.

For the treatment of onchocerciasis, the dosage of oral tablets as a single dose is based on body weight; however, the average dosage recommendation for adults and children weighing 15 kg or more is 150 µg/kg body mass. To treat threadworm infections, the recommended dosage is 200 µg/kg for adults and children weighing 15 kg or more as a single dose [50].

Topical administration of IVM in the dosage form of a 1.0% cream, by means of single or double application, is effective in the symptomatic treatment of demodicosis infections in humans [1]. In 2012, the FDA approved IVM in the treatment of head lice in humans (from 6 months and older); the approved dosage form is a 0.5% IVM lotion [1]. Treatment for resistant dermatitis and rosacea involves topical 1.0% IVM cream.

IVM is currently used to treat filarial and parasitic infections at approved doses of 150–400 μg/kg [14]. Researchers are exploring higher doses (>400 μg/kg) for new indications like soil-transmitted helminthiasis (STH) and malaria control. Consequently, a fixed-dose regimen (rather than weight-based) is being evaluated to simplify mass drug administration and potentially co-formulation with other drugs, including mebendazole or albendazole, which are provided in fixed dosages [14]. A recent study found that fixed doses of 18 mg and 36 mg IVM regimens were considered safe and effective in adults. By evaluating fixed-dose regimens, the safety profile of high-dose IVM is assessed to support the expanded use thereof [14]. Registered IVM trade product(s) from a few different countries intended for human use are listed in Table 1 [89,90,91,92].

### 3.2. Investigational

In the absence of established treatment regimens early in the coronavirus disease of 2019 (COVID-19) pandemic, individuals and healthcare providers explored the use of existing medications as potential therapeutic options. This practice, known as drug repurposing, was considered a practical and time-efficient approach, particularly given the urgent need for accessible treatments for COVID-19 [94,95]. Repurposed drugs offer several advantages as they are generally more affordable and widely available than newly developed therapies. Additionally, their safety profiles, including potential side-effects and contraindications, are already well-documented through previous clinical use, thus facilitating their progression into clinical trials more rapidly than the traditional timeline required for the development of novel pharmaceuticals [95,96].

As a result, a wide range of drugs (including IVM) originally developed for other conditions were investigated for their potential to mitigate the symptoms of COVID-19, both in clinical trials and off-label use [96,97]. However, this caused a surge in public demand for veterinary IVM formulations, which are not intended for human consumption [49]. Moreover, regulatory authorities such as the United States Food and Drug Administration (FDA) and National Institutes of Health (NIH) issued warnings against the use of IVM for COVID-19 treatment, citing a lack of robust evidence and the potential risks associated with its use [98].

#### 3.2.1. Antiviral

During this period, one of the most interesting aspects of IVM was its prospective antiviral effects against the severe acute respiratory syndrome coronavirus 2 (SARS-CoV-2), especially since various studies highlighted its antiviral properties [7,99,100]. It was proposed that IVM has a potential two-fold mechanism of action against the SARS-CoV-2 coronavirus, either as prophylaxis or as treatment post-infection [101]. Pertaining to prophylaxis, IVM potentially prevents the virus from entering host cells by blocking the SARS-CoV-2 spike protein from binding to angiotensin-converting enzyme 2 (ACE2) receptors and inhibiting the transmembrane protease, serine 2 (TMPRSS2) protein [102]. Additionally, IVM reduces the production of pro-inflammatory cytokines [102] and reactive oxygen species (ROS) [103], thereby mitigating the cytokine storm. It was also implied that IVM could inhibit the viral replication process by blocking the main protease or 3-Chymotrypsin-like Protease (3CLpro) found in coronaviruses and preventing the transport of viral proteins to the nucleus [104]. Furthermore, IVM appeared to reduce the overexpression of Hypoxia-inducible factor 1-alpha (HIF-1α), induced by the accessory viral protein ORF3a and promote the polarization of M2 macrophages, which could help alleviate the pro-inflammatory state. While these potential prophylactic mechanisms of actions suggested that IVM might be useful in treating COVID-19, further research was required to confirm its effectiveness [100] and refine its appropriate dosage requirements.

In terms of treatment (post-infection), a recent in vitro study evaluated the efficacy of IVM against SARS-CoV-2, where the results showed that 72 h of IVM treatment reduced viral load by 99.8% in Vero cells strongly expressing human signaling lymphocyte activation molecules (Vero/hSLAM cells) infected with SARS-CoV-2. These findings relate to the off-label use of IVM as a possible therapeutic option for COVID-19 [105]. However, the therapeutic doses required to achieve this antiviral effect were unobtainable since the minimum required dosages were higher than that approved by the FDA [94,100], which is 150–200 µg/kg (0.150–0.200 mg/kg) in a single oral dose [50]. Alternatively, Ceballos et al. [106] described a weight-based dosing regimen of 50–400 µg/kg for any person ≥ two years of age. Even though the suggested mechanisms directed against COVID-19 infection were promising, they are still largely inconclusive and require further research to determine whether the drug’s application in the treatment of these patients will be beneficial [94].

Apart from impacting SARS-CoV-2 infections, IVM suppresses viral replication in a wide range of flaviviruses, such as yellow fever [107], tick-borne encephalitis, Japanese encephalitis and dengue [7] via the potential targeting and inhibition of a viral helicase [7,107]. It is crucial to remember that IVM’s main target in mammalian cells is host protein, essential for intracellular transport, rendering it a host-directed agent (HDA); hence, IVM does not target a viral component directly. However, IVM comprises broad-spectrum activity against several different ribonucleic acid (RNA) viruses in vitro because it is an HDA. HDA’s reduce the viral load by inhibiting a key cellular process needed by the virus to create an infection. Consequently, the HDA prevents the virus from suppressing the host’s antiviral response and therefore, low doses administered during the early phase of infection can empower the body’s immune system to commence an antiviral response and reduce viral load before the infection yields control [108].

Research presented by Kaur et al. [109] investigating the potential applications of IVM beyond antiparasitic activity revealed that IVM presents antiviral properties by targeting the importin (IMP)-α/β1 interface, which is used by viruses to transport themselves into the nucleus and take over host activity. This mechanism allows IVM to potentially inhibit the replication of various viruses, including human immunodeficiency virus type 1 (HIV-1), dengue, zika, West Nile virus, Venezuelan equine encephalitis virus, chikungunya, and SARS-CoV-2 [109]. IVM’s ability to perform as a vigorous broad-spectrum specific inhibitor of IMP-α/β-mediated nuclear transport contributes to IVM’s function to block nuclear trafficking of viral proteins and consequently exhibits antiviral activity against several RNA viruses [7].

Human adenoviruses typically cause mild symptoms but can lead to severe disease in immuno-compromised individuals and children. Currently, there is no effective antiviral treatment for adenovirus-related ailments. However, research suggests that IVM may be a potential treatment option. Kaur et al. [109] further supports that IVM could potentially inhibit human adenovirus C5 (HAdV-C5) early gene transcription, genome replication, and protein expression, reduce the production of infectious viral progeny in a dose-dependent manner, suppress genomic replication of human adenovirus B3 (HAdV-B3), but not human adenovirus E4 (HAdV-E4) and interfere with the binding of viral E1A proteins to IMP-α without affecting the IMP-α/β1 interaction. These findings, however, were demonstrated in vitro, and further in vivo studies are needed to explore the efficacy of IVM in treating human adenoviruses and other viral conditions.

Pérez et al. [110] investigated the antiviral effects of IVM against Varicellovirus bovinealpha 1 (BoAHV-1), a virus that causes respiratory disease in cattle. They found that IVM showed significant antiviral activity against BoAHV-1 in two cell lines (Madin-Darby Bovine Kidney (MDBK) cells and bovine turbinate (BT) cells) at concentrations of 1.25, 2.50, and 5.00 µM, even the lowest dose of IVM (1.25 µM) reduced virus titers in both cell lines, with slight cytotoxicity that was only observed at the highest dose (5.00 µM) and the antiviral effects of IVM were evident within a range of concentrations achievable through therapeutic in vivo administration. These findings suggest that IVM may be a potential therapeutic agent for managing BoAHV-1 in infected cattle. However, further in vivo trials are needed to validate these results and explore the potential utility of IVM in treating BoAHV-1 infections. Nevertheless, the emerging antiviral mechanisms of IVM offer promising potential for future research.

#### 3.2.2. Antibacterial

Previously, the avermectin family was believed to possess no antibacterial properties [10,12,111,112,113,114]. However, data emerged in 2012 indicating that IVM could (at doses suitable for treating ocular or sexually transmitted disease (STD) infections) inhibit the bacterial pathogen *Chlamydia trachomatis* from infecting epithelial cells [7]. Investigations have revealed in vitro actions of IVM against *Mycobacterium ulcerans*, which is a bacterium responsible for causing Buruli ulcers [115]. Minimum inhibitory concentration (MIC) testing, time-kill assays and bioluminescent kill curves were conducted by Omansen et al. [115]; subsequent results indicated that IVM can inhibit the growth of various *M. ulcerans* strains and is also able to achieve dose-dependent killing of *M. ulcerans*. IVM possesses a bactericidal effect on strain growth inhibition that efficiently treats most drug-resistant and laboratory strains of *Mycobacterium tuberculosis* [115], thereby suggesting that IVM can be repurposed as a supplementary treatment of tuberculosis [7].

Furthermore, IVM demonstrated potent antibacterial activity against two out of twenty Gram-positive *Staphylococcus aureus* isolates through the curbing of biofilm formation [108,116]. This bacteriostatic effect involves the destruction of the bacterial cell wall and then attaching to bacterial deoxyribonucleic acid (DNA) after permeating the cell membrane, which in turn prevents biofilm formation of methicillin-resistant *S. aureus* (MRSA). Biofilms are multi-layered communities of bacteria that produce an increased resistance against host defenses and antimicrobial drugs. Biofilm communities lower the metabolism of cells, which renders it challenging to eliminate and consequently leads to treatment failure and recurrent infections [117]. Anti-staphylococcal properties of IVM against MRSA and methicillin-sensitive *S. aureus* (MSSA) isolates have been noted by Ashraf et al. [111]. In all the priorly mentioned studies, however, the in vitro antibacterial effects necessitated higher concentrations of IVM relative to its antiparasitic activity. This regrettably reduces the likelihood of using IVM as a potential antibacterial treatment remedy, since neurotoxicity might occur at these high dosages [7].

#### 3.2.3. Anticancer

IVM can potentially be used as an anticancer agent, due to its ability to downregulate antitumor targets in cancer stem-like cells (CSCs). Moreover, IVM possesses the ability to induce oxidative stress and mitochondrial dysfunction within cells, resulting in the selective killing of chronic myeloid leukemia cells, which contributes to the potential of IVM as an anticancer agent for patients diagnosed with leukemia [64]. IVM has also demonstrated the ability to cause intracellular chloride flux in human leukemia cells (in vitro) in effect aiding in cancer therapy [118,119]. Programmed cell death patterns have been noted with IVM administration, as the main type of programmed cell death induced by IVM is apoptosis. IVM is mainly able to induce apoptosis via the mitochondrial pathway, where the mitochondrial membrane potential is reduced and subsequently, cytochrome C is released from the mitochondria into cytoplasm [16].

Moreover, IVM may induce immunogenic cell death in cancer cell lines by modulating several pathways, such as (1) the Wingless signaling (WNT)-T cell factor (TCF) [16,120,121], (2) Hippo factors [116,118], and (3) protein kinase B (AKT)/mammalian target of rapamycin (mTOR) signaling pathways [16,122,123,124] as well as (4) inducing caspase-dependent apoptosis [116,118] and (5) p21-activated kinase 1 (PAK1)-mediated cytostatic autophagy [122,125,126,127]. Additionally, IVM functions as an activator of chloride channel receptors [16,108], an RNA helicase [16,128], a small-molecule mimetic of the surface-induced dissociation (SID) peptide [16,129], and an inducer of oxidative stress and mitochondrial dysfunction [16,130]. This consequently influences the proliferation and growth of malignant cells [16]. IVM targets tumor angiogenesis through the stimulation of apoptosis in human brain microvascular endothelial cells [16], promotes the reversal of drug resistance by suppressing multi-drug resistance (MDR) proteins [16] and has strong anti-mitotic activity, together with the ability to inhibit CSCs, particularly in breast cancer [16]. Furthermore, Sulik et al. [1] reported that IVM reduces the amount of stem cells that are involved in the progression, metastasis, and recurrence of cancer [1] and could reach the clinically significant concentration levels required to hinder the growth of tumors in humans [1]. Hence, IVM exhibits antitumor effects and may therefore benefit cancer patients [123].

In glioma cells, IVM induces apoptosis by stimulating caspase-3 and -9 and enhancing tumor protein p53 and Bcl-2 associated X protein (Bax) expression [100]. Additionally, it promotes autophagy in melanoma cells through ROS signaling pathways, leading to cellular self-destruction [100]. IVM also stimulates cell death in porcine trophectoderm and uterine luminal epithelial cells by disrupting calcium ion balance, mitochondrial membrane potential and generating ROS [100]. Furthermore, IVM inhibits hypoxia-inducible factor (HIF)-1α, a key factor in drug resistance, by blocking its translocation to the nucleus [100]. These findings suggest that IVM could be a valuable target for cancer treatments, particularly in combination with other therapies, warranting further research to confirm its efficacy and safety in humans.

#### 3.2.4. Anti-Inflammatory

Studies have confirmed that IVM facilitates the blockage of cytokine production by means of challenged lipopolysaccharide macrophages, consequently attributing advanced anti-inflammatory properties to this drug [131]. Furthermore, the literature states that it exhibits anti-inflammatory qualities specifically in T cell-mediated skin diseases [28]; hence, topical IVM preparations for the treatment thereof can be of great significance [64].

Conducted research utilized topical IVM to treat a range of inflammatory skin disorders, such as allergic dermatitis and perioral dermatitis, which displayed effective reductions in inflammation. IVM reduced inflammation by a suggested mechanism to minimize the generation of inflammatory cytokines and a reduction in the activation of T-cells specific to allergens. This was achieved by IVM’s ability to inhibit mitogen-activated protein kinases (MAPK) phosphorylation, c-Jun N-terminal kinase (JNK) and extracellular signal-regulated kinase 1 and 2 (ERK 1/2); consequently, resulting in the suppression of the inflammatory mediators (including nitric oxide (NO) and prostaglandin E2 (PGE2)) by the reduction in nitric oxide synthase (NOS) and cyclooxygenase-2 (COX_2_) gene expression [116].

#### 3.2.5. Other

Early investigations found that the administration of high IVM dosages resulted in enhanced chloride conductance in mammalian neural cells. In such cases, high-dose IVM has been effective in treating severe muscle spasticity in individuals with spinal cord injuries [132]. According to Sia et al. [9], IVM demonstrates wound healing activity by modulating the inflammatory process [133], transforming growth factor-beta 1 (TGF-β1) [134] and vascular endothelial growth factor (VEGF) levels. The combined antibacterial and anti-inflammatory activities of IVM can contribute to wound healing effects [9], especially since the antibacterial effect of IVM will prevent bacteria from colonizing within the injured area [135]. A recent study performed by Tian et al. [134] revealed that by investigating the mRNA and protein expression levels, IVM inhibited the proliferation of hypertrophic scar fibroblasts and drastically reduces α-smooth muscle actin, type I collagen, and cellular communication network factor 2 production. This indicates that IVM might be a potential therapeutic agent for the reduction in scar formation. Additionally, an in vivo study discovered that IVM promotes the regeneration of peripheral nerves when administered locally to the dermal wound site, which can be attributed to the inducement of fibroblasts to adopt a glia-like phenotype by upregulating neuronal and glial markers during the healing process [133]. Consequently, IVM creams at low doses possess the capability to treat parasite-infested wounds with minimal formation of scar tissue. Moreover, some studies have reported that IVM creams (at low dosages of 0.03–1.00%) reduced wound macroscopic indices (including hyperemia, edge oedema, exudation, and granulation tissue deposition) and increased wound healing rate, contraction rate and hydroxyproline deposition [9,111,136]. IVM creams are considered non-irritating and non-toxic to the skin up to concentrations of 10.00% (weight per weight (*w*/*w*)) [9].

Sulik and collaborators [1] assessed the insecticidal activity of subcutaneously administered IVM at a dose of 200 µg/kg or 400 µg/kg in hamsters to ward against sandfly vectors that spread leishmaniasis. Subsequent results showed promising mortality rates of the sandflies that fed on IVM-treated hamsters. As such IVM could be considered as a possible mode of leishmaniasis prevention [1].

IVM also demonstrates antimalarial activity and has been reviewed as experimental therapy against malaria. Studies have shown high mortality rates in varying species of malaria mosquitoes after they have fed on IVM-treated animal models [1].

The potential target conditions and diseases proposed by Crump [6] include myiasis, trichinosis, American- and African trypanosomiasis, asthma, and epilepsy, along with broader disease categories such as neurological, metabolically related, and Farnesoid X receptor (FXR)-mediated diseases. Since several of these (i.e., myiasis, American- and African trypanosomiasis) involve vector transmission, vector control measures may also be relevant.

### 3.3. Ivermectin Toxicity

#### 3.3.1. Ivermectin Side-Effects and Overdose Toxicity in Humans

Lethal dose 50% (LD_50_) is a standard toxicological metric that represents the dose of a substance required to cause death in 50% of a test population, typically expressed in milligrams per kilogram of body weight (mg/kg). It is commonly used to estimate acute toxicity and compare the relative toxic potential of chemical or pharmaceutical agents [137]. The exact LD_50_ of IVM in humans is not precisely known; therefore, data is derived primarily from animal studies due to ethical constraints in human testing. In preclinical models, the LD_50_ of orally administered IVM has been reported as approximately 25 mg/kg in mice and around 80 mg/kg in dogs [118]. These figures correspond to an estimated human-equivalent LD_50_ range of approximately 2.02 to 43.24 mg/kg, depending on the method of interspecies scaling used [118].

For context, this range is substantially higher than the FDA-approved therapeutic dose for humans, which is a single oral dose of 0.150 to 0.200 mg/kg for the treatment of specific parasitic infections [50]. However, in controlled clinical settings, IVM has demonstrated a wide safety margin, with doses up to 2 mg/kg administered in research environments showing only mild, transient adverse effects [14,138]. Overall, when used as directed, IVM is considered to have a favorable safety profile. However, at supratherapeutic doses, neurological symptoms such as ataxia, confusion and in severe cases, seizures may occur [123,139].

Moreover, studies have found that high-dose IVM (up to 0.800 mg/kg) compared to standard doses (0.200–0.400 mg/kg) in treating parasitic infections showed no significant increase in adverse effects with increased dosages and most of these adverse effects were mild to moderate and transient [14]. However, visual disturbances (although rare) were more prevalent when using higher doses of IVM. Regardless, it is still suggested that there is no significant relationship between the dose and severity of adverse effects, but rather that the safety profile of IVM is generally dependent on the type and severity of underlying conditions [14], which are more important factors to consider during safety determination. Underlying conditions, including disease-related lesions [140], microfilaremia (presence of microfilariae in the blood) [140], onchocerciasis [141,142,143,144], lymphatic filariasis [145] and Loa loa infections (eye worm) [142] are all associated with a higher risk of severe adverse effects with IVM treatment [14].

Mydriasis, vomiting, ataxia, lethargy, transient blindness, and tremors are some of the clinical indications of IVM toxicosis [139], which can quickly develop into neurotoxicity [123], including respiratory failure, stupor, seizures, coma, and death [139]. Usually, mydriasis is the first clinical indicator of overdose and the last symptom of IVM toxicity to subside [139]. Another possible adversity that may occur after administering IVM at a normal therapeutic single dose of 0.050–0.400 mg/kg [106] is the Mazzotti reaction. The Mazzotti reaction is stimulated by an immune response to dead microfilariae present in the patient when treated with IVM. Common clinical signs of this reaction include fever, rash, tachycardia, swelling of the lymph nodes and eye inflammation [17].

Acute overdose, increased serum levels after prolonged treatment, or genetic vulnerability can all contribute to IVM-induced toxicity [139]. IVM toxicosis lacks a specific antidote; however, activated charcoal may be given repeatedly in the event of an acute oral overdose in an attempt to prevent enterohepatic reabsorption. Intravenous lipid emulsion therapy has also proven effective in managing adverse reactions to lipophilic medications (such as IVM), though its success generally depends on discontinuing the medication along with supportive care. The clinical effects should then subside in a few days to weeks (depending on how severe the symptoms were). Additionally, physostigmine (a reversible cholinesterase inhibitor and parasympathomimetic alkaloid) has also been shown to improve neurological toxicity symptoms in the short-term. However, because of its significant cholinergic effects and acute activity, it is not advised for chronic use [139].

The target population carrying a frame shift deletion mutation in the ATP Binding Cassette Subfamily B Member 1 (ABCB1) gene (previously known as the multi-drug resistance gene, mdr1), which produces P-glycoprotein (P-gp; an adenosine triphosphate (ATP)-dependent transmembrane transporter protein) that performs a crucial function in the blood–brain barrier, is susceptible to IVM sensitivity. The deletion mutation results in severely shortened, nonfunctional P-gp molecules by causing the P-gp production to terminate prematurely. As a result, some medications’ ability to exit the central nervous system (CNS) is compromised, which causes a build-up of the drug (toxic levels) inside the CNS. Since IVM is one of P-gp’s substrates, the target population that is homozygous for this autosomal recessive gene exhibits the IVM-sensitive phenotype [139].

Research surrounding IVM toxicity has significantly contributed to its safe use in various applications. The following are findings and contributions of note:

Establishing safe dosage ranges: Toxicity studies have helped determine the maximum tolerated dose and safe dosage ranges for IVM in both humans and animals, lowering the risk of adverse effects. However, IVM has a weak potential for long-term toxicity with a wide margin of safety between therapeutic doses and toxic doses. Despite this, the emphasis on the importance of continued monitoring and research to ensure safe use remains crucial [105].

The acceptable therapeutic dosage ranges for IVM use in humans are 0.150–0.200 mg/kg (single oral dose every 12 months) or alternatively, 0.050–0.400 mg/kg for any person two years or older in age [106]. Research on IVM’s pharmacokinetic properties such as absorption, distribution, metabolism, and excretion (ADME) has informed dosing regimens and reduced the risk of toxicity [138,146,147]. Furthermore, identifying potential drug interactions and enhancing monitoring and management of toxicity is crucial; studies have revealed potential interactions between IVM and other medications, prompting healthcare providers to take precautions and adjust treatment plans accordingly. Drug interactions should also be considered prior to administering IVM to animals, since certain interactions increase systemic exposure to IVM, particularly within the CNS of animals, thus increasing the risk of neurotoxicity [55,148]. These drugs include ketoconazole [44,55], itraconazole [55], cyclosporine [55,149], erythromycin [55,150], amiodarone [55], and nifedipine [148,151]. In addition, it is known that alcohol, grapefruit, and orange juice metabolically interact with IVM, with IVM exhibiting noticeably higher plasma concentrations when given in conjunction with alcohol and grapefruit juice, while lower plasma concentrations are evident when consumed with orange juice [116]. Research has provided development of strategies for monitoring and managing IVM toxicity, including the use of biomarkers and treatment protocols for overdose or adverse reactions.

Toxicity research has led to the establishment of guidelines for IVM use in vulnerable populations, such as pregnant women [1,14], children, and individuals with liver or kidney disease [105]. The guidelines for IVM use in said individuals are summarized as follows [138,152,153]: for pregnant and breastfeeding patients, use should be avoided particularly in the first trimester. However, the normal therapeutic dose (0.050–0.400 mg/kg single dose) [106] may be administered under medical supervision as low concentrations of IVM have been detected in human breast milk. Current manufacturer guidelines advise that treatment during lactation should only be considered when the potential risk of delaying therapy in the mother is deemed greater than the potential risk to the nursing infant [154]. The approved IVM single oral dosages of 0.200 mg/kg for the treatment of strongyloidiasis and 0.150 mg/kg for the treatment of onchocerciasis are suitable for use in children weighing ≥15 kg [155]. Current reviews of safety data for children under 5 years of age or weighing less than 15 kg, who received IVM at approximate doses of 0.200 mg/kg for various infections, have not revealed any significant safety issues. Regardless, treatment decisions for children in this category should be made in consultation with a qualified healthcare provider [155]. No formal guidelines are stipulated for individuals with liver and kidney diseases, but close monitoring for toxicity is required for individuals with liver cirrhosis [156,157,158,159]. Moreover, IVM is typically regarded as safe for individuals diagnosed with porphyria, including the acute subtypes. Nevertheless, its use should be approached with clinical discretion, considering the patient’s comprehensive medical background and present condition. Prior to initiating therapy, it is advisable to seek guidance from a healthcare provider with expertise in the management of porphyria disorders [160].

Current uses (parasitic infections, such as onchocerciasis or river blindness [16,18] and research on IVM’s safety profile has enabled its use in new therapeutic applications, for example viral infections [7,99,100] like COVID-19, inflammatory diseases [64,99,100,161], bacterial infections [108,116], cancer treatment [28] and wound healing [9,133,134]. Finally, the understanding and establishment of IVM’s toxicity has led to the development of improved formulations, such as topical and transdermal formulations, which reduce systemic exposure and minimize adverse effects [162]. By elucidating the toxicological profile of IVM, appropriate dosage form development can optimize its use, minimize risks, and expand its applications, ultimately benefiting public health and improving patient outcomes.

#### 3.3.2. Safety Considerations for Humans Using IVM-Treated Animal Products

When used according to regulatory guidelines from bodies like the FDA, EMA [163], and Codex Alimentarius [164], including strict adherence to species-specific withdrawal periods (e.g., 49 days for subcutaneous injection in cattle under EU standards), IVM residues, marked by 22,23-dihydroavermectin B1a, typically deplete below established maximum residue limits, such as Codex values of 10.0000 µg/kg in cattle milk and 100.0000 µg/kg in liver and fat, or FDA tolerances of 1.6 ppm in cattle liver and 10.0 ppb in milk, ensuring minimal human health risks [165]. However, off-label or non-compliant use can result in exceedances, particularly in lactating dairy cattle where approval is often absent due to prolonged milk excretion, as seen in small-scale farms with milk residues averaging 12.4600 µg/kg and ranging up to 440.0000 µg/kg [166]. Regardless, dietary exposure assessments indicate low chronic risks, with estimated daily intakes (0.0200–0.0935 µg/kg body weight/day) [166] falling below the acceptable daily intake of 0.0000–10.0000 µg/kg body weight/day [164]. Residue depletion varies by species and product: quick in meat and liver from cattle, sheep, and swine (e.g., below 2.0000 µg/kg in swine tissues by 21 days post-0.4 mg/kg dose), but prolonged in poultry eggs (up to 71-day persistence, leading to impractical 57–102-day withdrawals), rendering it unsuitable for laying hens [163]. While excessive residue accumulation could theoretically cause neurotoxicity, real-world compliant exposure remains negligible, though caution is advised for sensitive groups like children with high consumption; ongoing surveillance and compliant sourcing are essential to mitigate emerging resistance and non-compliance issues.

## 4. Novel Dosage Form Development for Human Use

**Figure 5:**
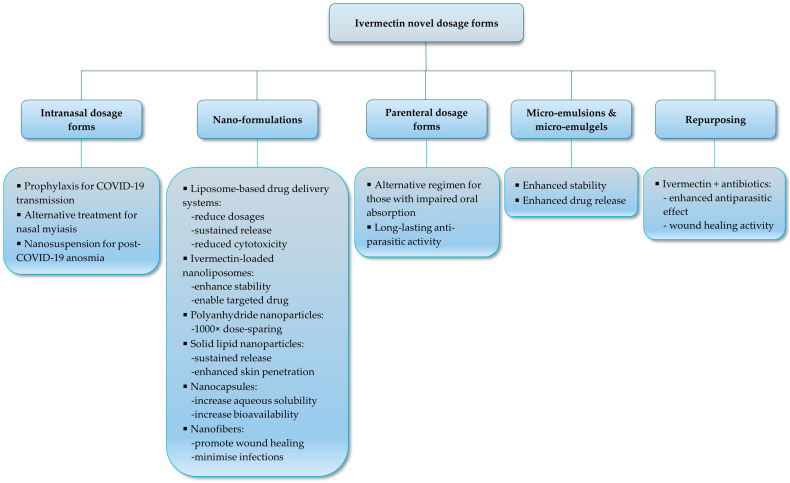
Schematic diagram illustrating some of the latest novel research conducted surrounding ivermectin’s potential for repurposing and formulation into unconventional API delivery systems.

The high lipophilicity and large volume of distribution of IVM present a complex challenge for the development of dosage forms. It is essential to explore various administration routes and innovative formulation strategies to effectively treat endo- and ectoparasitic infections in both humans and animals, despite the superior antiparasitic activity of IVM [167]. Several studies have investigated alternative IVM formulations and routes of administration, focusing on optimizing its therapeutic efficacy, safety, and bioavailability, as depicted in Figure 5. These advances in pharmaceutical technology have resulted in the development of targeted, innovative IVM dosage forms that potentially offer distinct pharmacological advantages, including enhanced therapeutic efficacy, improved patient compliance, and reduced adverse effects related to adipose tissue accumulation and variable bioavailability.

However, when comparing IVM formulations used across different species, some overlapping drug delivery strategies emerge along with species-specific challenges. For example, while oral solutions are effective for both humans and animals, factors such as metabolism, gastrointestinal conditions, and behavioral differences (like voluntary swallowing in humans versus involuntary swallowing in livestock) influence the choice and design of dosage forms [168]. Importantly, veterinary formulations often prioritize safety, stability, mass dosing, and cost-effectiveness, while human applications emphasize patient compliance, safety, and regulatory approval [169,170].

As a relevant example, innovations in nanomedicine have primarily been driven by research in human pharmaceuticals, but there is a growing adaptation of these advancements for veterinary use particularly for high-value animals or in controlling zoonotic diseases [171], whereas despite the superiority of nanomedicine demonstrated by academic innovations, clinical translation of nanomedicine for human use is impeded by a lack of regulatory guidelines for its safety and registration [172]. Additionally, the formulation strategies employed in human medicine have frequently preceded veterinary applications, although species-specific considerations remain crucial [171]. Recent advancements in dosage forms incorporating IVM have significantly enhanced the drug’s pharmacological performance, addressing longstanding issues related to solubility and bioavailability [66]. In this section, this review will highlight innovative dosage form development aimed at improving targeted and optimized delivery of IVM in veterinary and human medicine.

### 4.1. Liquid-Based Dosage Forms

Liquid-based dosage forms have evolved from solutions and suspensions into innovative vehicles by utilizing excipients such as ionic liquids for tailored drug delivery approaches [173]. Importantly, liquid-based drug delivery systems hold advantages such as solubilizing drugs prior to oral administration. Solutions have shown benefit in humans, where a study by Ceballos et al. [106] reported that high-performance liquid chromatography (HPLC) analysis of blood samples (retrieved 2–48 h post-treatment with IVM) showed an oral solution of IVM achieved notably higher systemic bioavailability (analytical peak area (AUC) = 1653 ngh/mL) than solid oral IVM formulations (tablet, AUC = 1056 ngh/mL; capsule, AUC = 996 ngh/mL) in healthy human adult volunteers, thereby eliminating the rate-limiting step of dissolution within the gastrointestinal tract before drug absorption can occur.

These findings underline the importance of formulation and dosage form development in determining therapeutic outcomes, particularly in species where gastrointestinal physiology may limit drug dissolution [174]. With regard to veterinary settings, Mestorino et al. [175] compared the pharmacokinetic profiles of a liquid IVM dosage form (solution) and a solid IVM dosage form (tablet) after oral administration to sheep. They concluded that the absorption half-life for the tablet was double the duration of that of the solution, confirming the higher rate of drug absorption by liquid dosage forms than solid dosage forms [175]. Additionally, the versatility of formulations such as solutions and suspensions enables their administration through various routes in both human (oral, topical, otic, ocular, nasal, parenteral, etc.) [176,177] and veterinary (oral, parenteral, topical, otic, ocular, etc.) medicine [55]. Liquid-based dosage forms also present simplified administration options for the pediatric population and livestock.

Moreover, these dosage forms have demonstrated superior mucoadhesion, muco-penetration, and spreadability at mucosal surfaces, protecting the ocular-, nasal-, buccal-, sub-lingual-, and vaginal epithelia, leading to targeted drug delivery [178]. As an example, a study by Errecalde and co-workers (2021) [167] reported that an IVM nasal spray attained elevated concentrations of IVM in the lungs and nasopharynx, accompanied by low systemic levels of IVM following intranasal administration. This work aimed to develop a nasal spray suitable for reaching tissues known for SARS-CoV-2 entry and replication, such as the nasopharynx, as a prophylactic treatment option to facilitate viral inhibition during early stages of the infection. This nasal spray delivers 1 mg of IVM per nostril, and repeated administration in 12 h intervals rendered significantly higher levels of IVM in the lung and nasopharynx target tissues, thereby demonstrating that this nasal spray can be a safe and effective alternative to orally administered IVM tablets [167]. As intranasal drug administration can be used for direct nose-to-brain delivery, IVM entry into the CNS can inflict IVM-related toxicity. Therefore, this study evaluated the safety of this nasal spray in a pig model with no adverse effects, neurotoxicity, serum biological, hematological, and histopathological changes at target tissues reported. Interestingly, the study noted that data variability between pig subjects existed, potentially due to the lack of control over the ventilator state of pigs during administration of the IVM nasal spray [167], whereas the argument can be made that humans can carefully follow nasal spray user directions to reduce dosing variability and achieve optimized positioning of nasal spray administration to avoid direct nose-to-brain delivery by aiming nasal drug administration at the respiratory pathway instead of the olfactory or trigeminal pathway.

### 4.2. Solid Oral Dosage Forms

Solid dosage forms are among the most widely used dosage forms for the oral route of administration due to their versatility, ease of administration, and ability to accommodate various drug delivery needs. Tablets are available in multiple forms, including standard swallowable tablets, chewable tablets, effervescent tablets, oral disintegrating tablets (ODTs), and modified-release formulations such as extended-release and enteric-coated tablets [179]. These variations enable tablets to be tailored for rapid, delayed, or sustained drug release, and for administration via oral, sublingual, buccal, rectal, and even vaginal routes [179]. In contrast, capsules, which include hard gelatin, soft gelatin (softgels), and modified-release types, are particularly suitable for encapsulating powders, granules, or liquids [180]. Though primarily administered orally, certain capsule forms can also be used rectally or vaginally for local or systemic effects [180]. Solid oral dosage forms containing IVM have been investigated in both human and veterinary medicine.

A phase I clinical trial was conducted by Muñoz et al. [181] involving 54 healthy adult human volunteers who received two experimental IVM treatments using a newly formulated 18 mg tablet, administered in fixed doses of 18 mg and 36 mg, and compared to the standard commercially available tablet with a weight-based dose of 150–200 μg/kg. Participants were divided into three groups based on body weight, and plasma IVM concentrations were monitored via HPLC for up to 168 h post-administration. Pharmacokinetic analysis indicated a t_½_ ranging from 81 to 91 h across the treatment groups. Both the 18 mg and 36 mg fixed-dose regimens demonstrated increased systemic exposure, measured by AUC_0t_ and C_max_, when compared to the reference weight-adjusted product.

These results support the pharmacokinetic rationale for fixed-dose IVM regimens, confirming both their safety and consistent drug exposure across a broad range of body weights [181]. Though generally more applicable to human use, some are also adapted for animals, particularly in companion animal medicine [55], where developing oral dosage forms with accurate dosing [182] and enhanced palatability are crucial to obtain optimal therapeutic efficacy and compliance with regard to both the animal and the owner [183]. Paul et al. [184] conducted two clinical trials (in Illinois and Florida) with Beagle dogs to evaluate the antiparasitic potency of an IVM chewable tablet, as well as two other IVM tablet dosage forms, against *Dirofilaria immitis* (heartworm) infections. They reported that all dogs tested negative for heartworm infestation using antigen assays and Knott tests 4.5 months after the trials began; IVM, administered at a dosage of 6 µg/kg across all three dosage forms, demonstrated 100% efficacy, indicating strong antiparasitic activity against the early developmental stages of *Dirofilaria immitis* [184]. Additionally, Canga et al. [185] stated that greater oral bioavailability in dogs is obtained with chewable tablet dosage forms relative to conventional tablets. Gogolewski et al. [186] conducted dose confirmation trials and field trials in Merino sheep and reported the successful development and antiparasitic efficacy of an oral IVM tablet against gastrointestinal nematode infections in the sheep.

Apart from the challenges associated with optimized oral IVM delivery in human adults and animals, the literature reports a dire need for safe and efficacious oral pediatric formulations to aid in neglected tropical diseases [187,188]. A recent study by Juan and co-workers [189] reported the development of an IVM ODT comprising a super disintegrant, diluent, lubricant, sweetening agent, and glidant. This study documented the development of porous tablets via direct compression, which was followed by sublimation as achieved by ammonium bicarbonate inclusion as a sublimating agent. The ODT formulation exhibiting the most favorable properties had a 16.9 s disintegration time, displayed a 2.8 Kp hardness measurement, and portrayed appropriate friability compliance. Additionally, pre-liminary in vivo evaluations confirmed rapid IVM absorption into the systemic circulation, with post-treatment systemic circulation uptake recovery reported for at least 25 h after administration in a rat model.

### 4.3. Powder Dosage Forms

Powder dosage forms are increasingly developed to aid in non-pulmonary and pulmonary diseases [190]. Powder dosage forms hold advantages such as superior stability compared to liquid-based dosage forms, presenting an attractive drug delivery option for protein and peptide delivery to bypass cold-chain management, and the literature reports prolonged residence time at mucosal surfaces leading to enhanced drug absorption [191]. Challenges related to the successful delivery of powders can be linked to particle size, powder flowability and dispersibility in aqueous media. Moreover, a holistic consideration of the drug-related aspects, such as the chemical structure of the drug, chemical stability, hygroscopicity, and the effective therapeutic dose of individual drugs, adds to the complexity of successful powder dosage form development [190]. This has given rise to sophisticated strategies of particle engineering to produce stable powders with desired properties such as improved aqueous solubility for orally administered powders and superior aerosolization performance to optimize pulmonary drug delivery [190,192,193].

Powder inhalation dosage forms for IVM have been explored to optimize targeted IVM delivery to the lungs as an alternative to oral IVM treatment in targeting SARS-CoV-2 infection. A study by Su and co-workers [96] reported the development of a spray-dried technique utilized to combine IVM and niclosamide dry powders, rendering an amorphous powder falling within the ideal range of 1–5 μm. The dry powder drug combination enhanced in vitro activity against SARS-CoV-2 (half maximal effective concentration (EC_50_) of 2.67 μM) compared to individual drug dry powders, revealing anti-SARS-CoV-2 in vitro activity for IVM (EC_50_ = 8.61 μM) and niclosamide (EC_50_ = 5.28 μM), respectively, thereby demonstrating the potential of dry powder preparations to mediate targeted pulmonary drug delivery to improve the therapeutic efficacy of IVM.

### 4.4. Topical Semi-Solid Dosage Forms

Semi-solid IVM dosage forms, both in human and veterinary medicine, have garnered scientific attention due to their localized effect following dermal and mucosal site application [194,195,196]. These dosage forms, including creams, ointments, and emulgels, offer benefits such as targeted drug delivery, reduced systemic side-effects, and improved therapeutic outcomes [197].

A recent study conducted by Aucamp et al. [198] investigated the topical and transdermal drug delivery potential of a cream, ointment, and emulgel containing IVM. Drug release studies found that IVM was effectively released from all three tested dosage forms. In vitro skin diffusion and tape stripping analyses demonstrated that only the ointment achieved detectable IVM concentrations for transdermal permeation. Moreover, the ointment showed the highest median IVM concentration in the epidermis-dermis (ED) and the second highest in the stratum corneum-epidermis (SCE), revealing it as an effective dosage form for topical IVM delivery. These results suggest that the ointment’s structural composition significantly contributed to its favorable release and diffusion characteristics, likely due to the absence of partitioning between phases, allowing IVM to be readily available for skin permeation [198]. The emulgel delivered the highest IVM concentration to the SCE and second highest to the ED, supporting its role as a dosage form suitable for topical IVM delivery [198]. In contrast, the cream demonstrated the poorest performance, with the lowest IVM concentration in the SCE and no detectable levels in the ED. This outcome indicates that insufficient release of the drug from the cream hindered effective skin penetration, emphasizing the importance of initial drug release in facilitating subsequent permeation steps [198]. These findings confirm that the dosage form type plays a critical role in determining drug delivery efficiency across the skin layers, while the cream and emulgel were categorized as topical formulations, only the ointment demonstrated successful transdermal IVM delivery [198]. Although therapeutic efficacy at transdermal concentrations remains uncertain, both emulgel and ointment may hold potential for rosacea treatment based on comparisons to systemic exposure achieved with the marketed product Soolantra^®^ [198]. These semi-solid dosage forms hold potential topical treatment alternatives for accelerated wound healing, eliminating demodex mites, treatment of scabies, peripheral neuropathy, rosacea, and neglected dermal tropical diseases such as Buruli ulcer [9,115,133,196,199,200].

### 4.5. Nanoformulations and Nanostructured Carriers

Nanoformulations enhance three key features of an ideal drug delivery system: superior targeting while controlling drug release and drug distribution [201,202]. This is of particular importance when treating viral infections and parasitic infestations, as the inability to maintain sufficient therapeutic drug levels can lead to resistance developing [24,203].

Importantly, nanoformulations can overcome the intrinsically low oral bioavailability of lipophilic drug entities which frequently challenge the effectiveness of conventional oral dosage forms. Referring to factors such as poor aqueous solubility, limited ability to cross the intestinal epithelium, degradation within the harsh gastrointestinal environment, and susceptibility to efflux mechanisms and cytochrome P (CYP) 450 mediated metabolism, which can collectively hinder consistent drug absorption and lead to unpredictable plasma concentration profiles [204].

With the increasing development of drugs exhibiting low membrane permeability, there is a growing focus on strategies aimed at improving both their intestinal uptake and overall systemic bioavailability. As such, a broad range of nano-drug delivery vehicles has been formulated to enhance the transport and absorption of drugs. The literature has indicated that careful selection of nano-drug delivery vehicles, along with precise adjustment of their physicochemical properties, can significantly improve drug absorption [204]. Beyond their ability to shield drugs from enzymatic degradation and acidic conditions in the gastrointestinal tract and to enhance intraluminal drug solubility, nanoformulations also facilitate more efficient transport across the intestinal barrier [204]. Among the most widely utilized nanoformulations are polymeric nanoparticles (PNPs), liposomes, micelles, niosomes, SLNs, nanostructured lipid carriers (NLCs), nano-emulsions, self-nano-emulsifying drug delivery systems (SNEDDS), nanocrystals, mesoporous silica nanoparticles (MSNs), and dendrimers [204]. Signifying that nanotechnology has emerged as a particularly promising avenue for improving IVM delivery due to its versatile tailorability.

Awad et al. [43] produced IVM nanocrystals intended for topical drug delivery using the microfluidization technique. In vitro results demonstrated a 24-fold increase in the drug dissolution rate compared to raw drug material, and in vivo studies indicated a 3-fold increase in dermal drug deposition relative to the raw drug material. In another study conducted by Velho et al. [47], IVM was encapsulated in two distinct nanoformulations: mesoporous silica particles (IVM-MCM), representing an inorganic platform, and poly(ε-caprolactone) nanocapsules (IVM-NC), a widely studied biodegradable polymer-based system. The IVM-MCM formulation exhibited well-defined hexagonal mesoporous architecture, reduced surface area, and a high drug loading capacity of 10% *w*/*w*. In contrast, the IVM-NC system displayed a mean particle size of 196 nm, complete encapsulation efficiency (100%), good physicochemical stability in aqueous dispersion, and a drug loading of 0.1% *w*/*w*. Despite these differing structural and compositional attributes, both nanocarriers significantly improved the aqueous solubility of IVM relative to its crystalline form. After 72 h in a dialysis setup, IVM-MCM and IVM-NC achieved drug release levels of 72% and 78%, respectively, while the crystalline drug exhibited only 40% release under the same conditions. Furthermore, the two formulations demonstrated distinct release behaviors: IVM-NC offered a more prolonged and controlled release profile throughout the experimental period compared to the IVM-MCM formulation. These findings underscore the importance of considering factors such as drug loading and release kinetics in the design and optimization of IVM nanoformulations. The comparative analysis, along with considerations of administration routes and safety for both human and veterinary applications, supports the strategic development of nano-drug delivery vehicles to facilitate clinical translation [47].

Nano-drug delivery vehicles are particularly valuable for both systemic and topical dosage form applications, offering opportunities for targeted drug delivery and controlled release [204,205]. In veterinary contexts, nanotechnology may support long-acting formulations for livestock whilst simultaneously preventing the incidence of parasitic resistance [206,207]. In terms of human medicine, nano-vehicles offer enhanced treatment for conditions like onchocerciasis and scabies, where consistent therapeutic levels are critical [43].

More complex nanostructured systems, including NLCs and dendritic nanoparticles, have been investigated for their potential to provide targeted and sustained IVM delivery. Xu et al. [208] investigated the use of NLCs to improve the potential antiviral efficacy of IVM against porcine epidemic diarrhea virus (PEDV), a highly pathogenic coronavirus in piglets. While IVM is an approved antiparasitic agent, its limited bioavailability restricts its effectiveness. The researchers developed IVM-loaded NLCs, which exhibited favorable physicochemical properties and high drug encapsulation efficiency [208]. In vitro cellular uptake and antiviral assays using coumarin-6 (C6) in Vero cells (obtained from African green monkey epithelial cells) revealed that the encapsulated IVM demonstrated enhanced cellular uptake and significantly greater inhibition of PEDV replication compared to raw material IVM. Moreover, the formulation also reduced virus-induced oxidative stress, mitochondrial damage, and apoptosis in infected cells [208]. These findings suggest the potential of drug repurposing of IVM using NLCs as a drug delivery vehicle to broaden the therapeutic scope of IVM beyond its antiparasitic activity [208]. Additionally, NLCs are relevant in topical and transdermal applications [209], especially regarding IVM, since enhanced skin penetration of the drug is desired for optimal therapeutic efficacy [198]. Dave and Krishna Venuganti [210] discussed the applicability of dendritic nanoparticles for improved transdermal drug delivery, as dendrimers enhance drug penetration by increasing the solubility of the drug and facilitating its partitioning and diffusion into the skin. These formulations may be highly beneficial in both human and veterinary settings, especially for scenarios where systemic exposure of IVM via the skin is preferable to conventional oral dosage form administration, to improve bioavailability and patient compliance, sustained drug release, and reduced gastrointestinal side-effects [211].

### 4.6. Lipid-Based Formulations

Lipid-based formulations have garnered significant scientific attention by exploiting the uptake of lipids from the gastrointestinal tract into the lymphatic system, thereby circumventing the first-pass hepatic metabolism [212,213,214]. The lymphatic system imperatively regulates immune system function, fluid homeostasis, and lipid metabolism as a unidirectional transport network functioning in parallel to the circulatory system. Hence, improving the dissolution of lipophilic drugs via lipid-based formulation inclusion provides the opportunity to enhance drug absorption as naturally mediated by the intestinal digestion response to metabolize an exogenous lipid carrying a drug [214]. For instance, self-emulsifying drug delivery systems (SEDDSs) and micelles have shown strong potential for increasing the bioavailability of hydrophobic drugs such as IVM [215,216].

A study conducted by Patel et al. [217] investigated the incorporation of IVM into a SEDDS to improve the drug’s low aqueous solubility and poor bioavailability, ultimately enhancing its oral absorption. The optimized solid SEDDS formulation, containing soybean oil, Tween^®^80, and Span^®^80, was converted into a solid dosage form using surface adsorption followed by encapsulation in hard gelatin capsules. In vitro dissolution tests and in vivo pharmacokinetic studies (using male Wistar rats), respectively, demonstrated significantly improved drug release profiles and approximately double the bioavailability compared to a standard oral suspension [217]. The findings suggest that solid SEDDSs are a promising lipid-based formulation to enhance the bioavailability of IVM following administration from orally administered solid dosage forms [217].

Importantly, the lymphatic uptake of IVM can allow for targeting of lymphatic filariasis, known to inflict lymphatic dysfunction and with the potential to lead to complications such as elephantiasis and irreversible lymphedema [218]. The literature reports that lymphatic filariasis requires a dose of 400 µg/kg of IVM compared to the 150 to 200 µg/kg to optimally treat strongyloidiasis, enterobiasis, and onchocerciasis [219,220], thereby signifying the importance of targeting parasitic infections via direct delivery to the lymphatic system to potentially decrease the dose needed to successfully eliminate lymphatic parasitic infestations, as higher IVM dosages are notoriously linked to a higher incidence of ocular side-effects [220].

## 5. Status of Clinical Trials with Novel Dosage Forms

Despite extensive searches across the scientific literature and databases (up to October 2025), no registered or published human clinical trials have been identified for novel nanoparticle formulations of IVM; apart from a few studies located in Egypt (trial NCT04723459, trial NCT04716569, trial NCT04951362), although the status of these trials remains unknown [221,222,223]. As such, research on novel IVM nanoformulations remains confined to preclinical stages including in vitro, ex vivo, and animal models. These formulations, such as poly(lactide-co-glycolide) (PLGA)-based nanoparticles for oral delivery [224], lipid-polymer hybrids for inhalation [225], and nanostructured lipid carriers for topical application [226], aim to enhance IVM’s poor aqueous solubility, bioavailability, and targeted delivery for applications in antiparasitic, antiviral, anti-inflammatory, and anticancer therapies. Preclinical studies demonstrate improved encapsulation efficiencies (68–80%), sustained release profiles, and superior efficacy compared to free IVM, such as reduced viral entry or enhanced lung deposition in models; however, significant challenges persist, including scalable production, biocompatibility, regulatory hurdles, and ensuring safety at therapeutic doses to facilitate clinical translation [227].

Recent studies [66,222,228,229,230] expand applications, for example IVM-loaded SLNs combined with albendazole show enhanced effects against trichinellosis in enteric phases, while nanoplatforms repurpose IVM for autophagy modulation in colorectal cancer chemo-phototherapy. A patent for nano-IVM compositions suitable for nebulization highlights potential for respiratory delivery during pandemics [231]. These formulations leverage encapsulation to protect IVM from degradation, enable controlled release, and facilitate targeted delivery. Preclinical data suggest 2–3-fold bioavailability improvements in rats, but human extrapolation remains unvalidated [232]. In contrast, conventional IVM has undergone clinical trials (e.g., SAINT trial NCT04390022) for the possible treatment of COVID-19, often at high doses with mixed efficacy and toxicity concerns [233]. Nanoparticles could bridge this gap by achieving therapeutic concentrations safely, however, translational barriers are multifaceted:

- Physicochemical issues: Achieving uniform particle sizes post-processing and complete drug release (often <60%) without agglomeration [232].
- Safety and biocompatibility: Potential immunogenicity from components like polyethylene glycol (PEG); narrow therapeutic window risking neurotoxicity at high doses [234].
- Pharmacokinetic hurdles: Overcoming barriers like gut epithelium or mucus clearance; validating enhancements in larger models [235,236,237].
- Regulatory and scalability: Good Manufacturing Practice (GMP)-compliant production, demonstrating superiority to approved forms, and navigating intellectual property (e.g., patents for nebulized nano-IVM) [231].
- Efficacy concerns: Addressing resistance in parasites and lack of human in vivo data for novel indications [227].

Future efforts should prioritize phase I trials, advanced models (e.g., humanized mice), and multifunctional designs integrating diagnostics. While the preclinical promise of novel dosage forms is evident, rigorous validation is essential for potential clinical application, particularly in urgent contexts like pandemics or parasite resistant infections.

## 6. Conclusions

Globally, IVM is one of the most frequently used antiparasitic agents due to its remarkable pharmacological activity against a variety of parasitic species [64]. Moreover, IVM has demonstrated highly promising repurposing potential [9,115,133,196,199,200]. Currently, IVM is available in numerous veterinary oral, topical, and subcutaneous dosage forms. Dosage forms intended for human consumption/application are restricted to a single administration, solid oral dosage form, and a dermal cream. However, the repurposing potential exhibited by IVM during the COVID-19 pandemic rekindled scientific interest in this versatile anti-parasitic agent, as evident from the numerous publications reporting advanced dosage form development to optimize targeted drug delivery and the therapeutic efficacy of IVM [167,189,198,208].

Importantly, IVM has a favorable safety profile and is well-tolerated by most mammals, including humans (given the appropriate dose), with a low incidence of adverse effects [7]. However, the occurrence of adverse effects is not always dose-dependent but can also correlate with underlying conditions such as the presence of dead microfilaria that leads to the Mazzotti reaction [17,238]. Therefore, an overdose involving IVM may lead to neurological dysfunction, together with other systemic symptoms, and can cause a coma or even be fatal [123]. Hence, the need to improve IVM’s bioavailability is deemed essential to enable the development of lower dose formulations while maintaining improved therapeutic outcomes [239,240]. Consequently, investigating transdermal IVM delivery is advisable, since it offers numerous advantages including avoidance of hepatic first-pass metabolism, improved patient compliance, and reduced risk of systemic adverse effects [241]. However, IVM lacks the ideal physicochemical properties needed to effectively permeate the skin. Therefore, attempts are needed to prompt efficient drug delivery, such as including penetration enhancers into dosage forms [242,243], optimizing nano-IVM delivery vehicles [244], improving IVM physicochemical properties via solid-state modifications [245,246,247], enhancing skin diffusion with microneedles [88], or incorporating IVM into lipid-based dosage forms to mediate the uptake of IVM via the dermal lymphatic system [248].

Another strategy to optimize IVM efficacy, as suggested by Su et al. [96], is the development of effective combination therapies. Carefully selected drug combinations have demonstrated superior antiviral efficacy against SARS-CoV-2 in vitro compared to individual agents, potentially due to synergistic effects that may also lower the likelihood of resistance development. Notably, the in vitro co-administration of IVM and niclosamide has shown synergistic activity against SARS-CoV-2 [96]. Therefore, delivering these agents concurrently and directly to therapeutic target sites may not only enhance their antiviral effectiveness in vivo but also help suppress the emergence of resistant viral strains [96].

## 7. Future Perspectives

Future dosage form development should aim to interlink innovation across both human and veterinary fields, focusing on enhancing efficacy, safety, and accessibility in both humans and animals. Continued comparative research, prompting multi-disciplinary and inter-disciplinary innovations, can bridge scientific knowledge gaps regarding animal, human, and environmental health concerns, as demonstrated by the COVID-19 pandemic. The literature refers to the One Health concept, which originated from the One Medicine notion supporting collaboration between scientific disciplines, veterinary, and human health sciences to successfully manage non-communicable and communicable diseases by solving health challenges burdening human and veterinary medicine. This approach leverages insights from other scientific fields to expedite development in a different area. This also suggests that despite differences between species, therapeutic advances can benefit both animals and humans by offering a useful foundation for regulatory translation of veterinary products to dosage forms suitable for human consumption, and vice versa, thereby creating a two-way beneficial exchange system to unlock the full potential of repurposing a drug as versatile as IVM [47,249,250,251].

## Figures and Tables

**Table 1:** Registered IVM products for human use.

Country	Company	Registered IVM Trade Product(s)	Dosage Form and Strength	Indication(s)
Australia	Galderma Australia Pty Ltd.	Vastreka	Topical cream, 1%	Rosacea
Canada	Pharmascience Inc.	PMS-Ivermectin	Topical cream, 1%	Rosacea
Canada	Galderma Canada Inc.	Rosiver	Topical cream, 1%	Rosacea
Canada	Merck Canada Inc.	Stromectol	Oral tablet, 3 mg	Intestinal strongyloidiasis, onchocerciasis, ascariasis, trichuriasis, ancylostomiasis, hookworm diseases, lymphatic filariasis, scabies
Canada, India, United States of America	Rubicon Research Ltd.	Ivermectin	Oral tablet, 3 mg, 6 mg	Intestinal strongyloidiasis, onchocerciasis, ascariasis, trichuriasis, ancylostomiasis, hookworm diseases, lymphatic filariasis, scabies
India	Zydus Lifesciences Global Fze	Ivermectin	Topical cream, 1%	Rosacea
India	Senores Pharmaceuticals Inc.	Ivermectin	Oral tablet, 3 mg	Intestinal strongyloidiasis, onchocerciasis, ascariasis, trichuriasis, ancylostomiasis, hookworm diseases, lymphatic filariasis, scabies
Israel	Padagis Israel Pharmaceuticals Ltd.	Ivermectin	Topical cream, 1%	Rosacea
Israel	Taro Pharmaceutical Industries Ltd.	Ivermectin	Topical lotion, 0.5%	Head lice infestations
South Africa, United States of America	Galderma Laboratories Lp	Soolantra *	Topical cream, 1%	Rosacea
United States of America	Teva Pharmaceuticals USA Inc.	Ivermectin	Topical cream, 1%	Rosacea
United States of America	Edenbridge Pharmaceuticals Llc	Ivermectin	Oral tablet, 3 mg	Intestinal strongyloidiasis, onchocerciasis, ascariasis, trichuriasis, ancylostomiasis, hookworm diseases, lymphatic filariasis, scabies
United States of America	Epic Pharma Llc	Ivermectin	Oral tablet, 3 mg	Intestinal strongyloidiasis, onchocerciasis, ascariasis, trichuriasis, ancylostomiasis, hookworm diseases, lymphatic filariasis, scabies
United States of America	Merck Sharp And Dohme Corp.	Stromectol	Oral tablet, 3 mg	Intestinal strongyloidiasis, onchocerciasis, ascariasis, trichuriasis, ancylostomiasis, hookworm diseases, lymphatic filariasis, scabies
United States of America, India, Ireland, Switzerland	Arbor Pharmaceuticals Llc	Sklice	Topical lotion, 0.5%	Head lice infestations

* According to a statement released by the South African Health Product Regulatory Authority (SAHPRA) in March of 2021, IVM is registered for human use in South Africa in the form of a 1% topical IVM cream with trade name Soolantra^®^. Soolantra^®^ is indicated for the localized treatment of moderate to severe inflammatory lesions caused by papulopustular rosacea in adults [93].

## Data Availability

Not applicable.

## Abbreviations

The following abbreviations are used in this manuscript:

IVM	Ivermectin
COVID-19	Coronavirus disease of 2019
FDA	United States Food and Drug Administration
WHO	World Health Organization
NIH	National Institutes of Health
GIT	Gastrointestinal tract
SARS-CoV-2	Severe acute respiratory syndrome coronavirus 2
ACE2	Angiotensin-converting enzyme 2
TMPRSS2	Transmembrane protease, serine 2
ROS	Reactive oxygen species
3CLpro	3-Chymotrypsin-like Protease
HIF-1α	Hypoxia-inducible factor 1-alpha
Vero/hSLAM cells	Vero cells strongly expressing human signaling lymphocyte activation molecules
HDA	Host-directed agent
RNA	Ribonucleic acid
IMP	Importin
HIV-1	Human immunodeficiency virus type 1
HAdV	Human adenovirus
BoAHV-1	Varicellovirus bovinealpha 1
MDBK	Madin-Darby Bovine Kidney
BT	Bovine turbinate
STD	Sexually transmitted disease
MIC	Minimum inhibitory concentration
DNA	Deoxyribonucleic acid
*M. ulcerans*	*Mycobacterium ulcerans*
*S. aureus*	*Staphylococcus aureus*
MRSA	Methicillin-resistant *Staphylococcus aureus*
MSSA	Methicillin-sensitive *Staphylococcus aureus*
CSCs	Cancer stem-like cells
WNT	Wingless signaling
TCF	T-cell factor
mTOR	Mammalian target of rapamycin
PAK1	p21-activated kinase 1
SID	Surface-induced dissociation
MDR	Multi-drug resistance
Bax	Bcl-2 associated X protein
HIF	Hypoxia-inducible factor
JNK	c-Jun N-terminal kinase
ERK 1/2	Extracellular signal-regulated kinase 1 and 2
NO	Nitric oxide
PGE2	Prostaglandin E2
NOS	Nitric oxide synthase
COX_2_	Cyclooxygenase-2
TGF-β1	Transforming growth factor-beta 1
VEGF	Vascular endothelial growth factor
FXR	Farnesoid X receptor
LD_50_	Lethal dose 50%
w/w	Weight per weight
ABCB1	ATP Binding Cassette Subfamily B Member 1
P-gp	P-glycoprotein
ATP	Adenosine triphosphate
CNS	Central nervous system
ADME	Absorption, distribution, metabolism, and excretion
SLNs	Solid lipid nanoparticles
Hb	Hemoglobin
TLC	Total leucocyte count
DLC	Differential leucocyte count
BUN	Blood urea nitrogen
ALT	Alanine transaminase
AST	Aspartate transferase
C_max_	Peak plasma concentration
t_½_	Elimination half-life
IV	Intravenous
T_max_	Duration/time to reach C_max_
SAHPRA	South African Health Product Regulatory Authority
HPLC	High-performance liquid chromatography
AUC	Analytical peak area
ODT(s)	Oral disintegrating tablet(s)
EC_50_	Half maximal effective concentration
ED	Epidermis-dermis
SCE	Stratum corneum-epidermis
CYP	Cytochrome P
PNPs	Polymeric nanoparticles
NLCs	Nanostructured lipid carriers
SNEDDS	Self-nano-emulsifying drug delivery systems
PLGA	Poly(lactide-co-glycolide)
PEG	Polyethylene glycol
GMP	Good Manufacturing Practice
MSNs	Mesoporous silica nanoparticles
IVM-MCM	Ivermectin mesoporous silica particles
IVM-NC	Ivermectin poly(ε-caprolactone) nanocapsules
PEDV	Porcine epidemic diarrhea virus
C6	Coumarin 6
SEDDS	Self-emulsifying drug delivery systems
